# Bruce’s Principles of Treatment

**Published:** 1900-06

**Authors:** 


### Books and Magazines.

---

BRUCE'S PRINCIPLES OF TREATMENT.—The principles of treatment and their application to the practice of medicine. By J. Mitchell Bruce, M. D., F. R. C. P., Lecturer on the Practice of Medicine in Charing-Cross Hospital, London; Examiner in Medicine, Royal College of Physicians, London. Revised to conform with the U. S. Pharmacopœia by E. Q. Thornton, M. D., Jefferson Medical College, Philadelphia. In one octavo volume of 625 pages. Cloth, $3.75, net. Lea Brothers & Co., Philadelphia. 1900.

This work is out of the ordinary, in that it seeks to simplify the subject under discussion by reducing it to its governing principles, the treatment. For instance, the author starts out by assuming no therapeutical laws, then proceeds to find them in the familiar facts of etiology, pathological anatomy and the clinical characters of disease. The reason for each constituent in a prescription is given, and it is based upon a scientific and clinical knowledge. This book should interest all classes of practitioners, and there are none who will fail to derive benefit from it by its study.

It is divided into two parts and thirty-two chapters. Part I is devoted to an introduction, giving the plan of the work, the principles of treatment founded on etiology, pathology, the clinical characters of disease, its course, the personal factor, etc. The means and the art of treatment are discussed at length.

Part II gives the illustrations of the principles of treatment, naming the diseases in order.

Altogether the work is valuable to all classes, and shows us how the English practice medicine. T. J. B.

